# Effects of gestational haloperidol exposure on mRNA expressions related to glutamate and GABA receptors in offspring

**DOI:** 10.1016/j.ibneur.2023.09.012

**Published:** 2023-10-02

**Authors:** Hiroshi Kumon, Yuta Yoshino, Yu Funahashi, Shinichiro Ochi, Jun-ichi Iga, Shu-ichi Ueno

**Affiliations:** Department of Neuropsychiatry, Molecules and Function, Ehime University Graduate School of Medicine, Shitsukawa, Toon, Ehime 791–0295, Japan

**Keywords:** Haloperidol, Pregnant, Glutamate receptor, GABA receptor

## Abstract

Antipsychotic treatment is vital for patients with schizophrenia even in the perinatal period, but the impact at the molecular biological level on offspring is unclear. The aim of the present study was to investigate the effects of intraperitoneal haloperidol injection to pregnant mice on glutamate and GABA receptors in the brain of offspring mice. Eight-week-old pregnant mice were treated with either intraperitoneal haloperidol or normal saline injection, and their offspring were defined as F1 mice. In addition, eight-week-old male mice were used as acute mice that were intraperitoneally injected with haloperidol or normal saline for 20 days. mRNA expression levels were measured by RT-qPCR. Western blotting was performed of the frontal lobes of F1 mice. In the hippocampi of F1 mice, Grik3 (p = 0.023) and Gabra3 (p = 0.004) mRNA expression levels were significantly higher in the haloperidol group than in the control group, whereas Gria2 (p < 0.001) and Grin2a (p < 0.001) mRNA expression levels were significantly lower in the haloperidol group than in the control group. Gria2 (p = 0.015), and Grik3 (p = 0.037), and Grin2a (p = 0.012) mRNA expression levels were significantly lower in the haloperidol group than in the control group in the frontal lobes of F1 mice. In the hippocampi of acute mice, Grik3 (p = 0.049) and Gabra3 (p = 0.007) mRNA expression levels were significantly decreased in the haloperidol group. Fetal exposure to haloperidol can affect glutamate and GABA receptors through mRNA expression changes in the brain of offspring.

## Highlights

• Haloperidol exposure in pregnancy raised Grik3 and Gabra3 mRNA levels, while reducing Gria2 and Grin2a in offspring hippocampi.
• Haloperidol exposure in pregnancy lowered Gria2, Grik3, and Grin2a mRNA in offspring frontal lobes.
• Prenatal haloperidol had no notable effect on offspring temporal lobe mRNA expression levels.
• Haloperidol-exposed offspring showed reduced GRIK3 protein in frontal lobes.

## 1 Introduction

Approximately 1 in 10,000 adults develops schizophrenia characterized by positive symptoms, negative symptoms, and cognitive dysfunction each year (Häfner and an der Heiden, 1997). Schizophrenia patients have over a two-fold risk of dying and, thus, a short life expectancy, approximately 20 years less than the general population (Auquier et al., 2007, Laursen et al., 2014, McGrath et al., 2008). Antipsychotic treatment is vital for schizophrenia patients to prevent relapse even in the perinatal period. However, antipsychotics could increase the risk of congenital malformations or withdrawal-emergent syndrome, including tremor, irritability, and somnolence (Coppola et al., 2007, Gentile, 2010). In this regard, some evidence is available at the phenotype level, but the impact at the molecular biological level on offspring is unclear.

The glutamate and dopamine hypotheses have been considered among the pathologies of schizophrenia. Those neurotransmitters are especially relevant to positive symptoms (Howes et al., 2015, Javitt, 2007, Javitt, 2010). Several studies have investigated the association of schizophrenia with glutamate receptor (Gardoni et al., 2008, Itoh, 1983, Kilic et al., 2010, Ziólkowska and Höllt, 1993). It has been suggested that one of the glutamate receptors, the N-methyl-d-aspartate (NMDA) receptor, is strongly related to symptoms of schizophrenia because phencyclidine, a noncompetitive NMDA agonist, induces emotional and cognitive deficits in schizophrenia (Javitt et al., 2012, Mouri et al., 2007, Nabeshima et al., 2006). Moreover, meta-analyses have reported that polymorphisms of *GRIK3*, the principal subunit of the kainate-type ionotropic glutamate receptor, increased schizophrenia risk by 30 % (Dai et al., 2014). Antipsychotics have been developed based on the various neurotransmitter hypotheses such as dopamine, serotonin, glutamate, glycine, cannabidiol, and estrogen (Maric et al., 2016, Stępnicki et al., 2018). On the other hands, it has been considered that the disability of GABAergic interneurons on the cerebral cortex related to the cognitive disfunction of schizophrenia (Inan et al., 2013). Previous studies show the relationship between schizophrenia and gamma-aminobutyric acid (GABA) receptor (Benes et al., 1992; Marques et al., 2021). In patient with schizophrenia, the mRNA expression that codes for the GABA receptor subunit changed in the dorsolateral prefrontal cortex (Beneyto et al., 2011).

Some first-generation antipsychotics including haloperidol (HAL) and many second-generation antipsychotics have been used to treat schizophrenia (Stępnicki et al., 2018). Although the main mode of action of HAL is as an antagonist of the D2 dopamine receptor (Nyberg et al., 1995), it also has antagonistic actions for the NMDA receptor (Zhuravliova et al., 2007) and the sigma-1 receptor (Dalwadi et al., 2017). Six-month HAL administration increased glutamine and total GABA levels in forebrain tissue in rats (Konopaske et al., 2013), and HAL induced neurotoxic effects resulting in neuronal death (Nasrallah and Chen, 2017).

It has been reported that the transplacental transfer rate of HAL was 65.5% in humans (Newport et al., 2007). In a cohort study, gestational HAL exposure increased the risk of fetal loss from 22.2% in the 3–6 months prior to conception to 78.6% with first trimester exposure (Boland et al., 2017). Although the transplacental transfer rate in the mouse is unknown, intraperitoneal HAL injection to pregnant mice induced recognition memory deficits and impaired the proliferation and maturation of adult-born dentate granule cells in offspring (Wang et al., 2019). With respect to intraperitoneal HAL injection to pregnant mice, HAL more easily leaves and enters into the brain in the fetal circulation because it contains less protein than the maternal circulation (Seeman, 2004). Moreover, it has been reported that mRNA expressions of dopamine D2 and D3 receptors in the prefrontal cortex (PFC), hippocampus, and nucleus accumbens were increased in adult rats treated by methylazoxymethanol acetate, a neurotoxin that reduces DNA synthesis, which is used as a schizophrenia model (Stark et al., 2020).

The aim of the present study was to investigate the effects of intraperitoneal HAL injection to pregnant mice on Grik, Gria and Grin, subunit of the glutamate receptor, and Gabra, subunit of the GABA receptors, in the brain of offspring mice. From in vivo studies of brain anatomy in patients with schizophrenia, a smaller cortical volume in frontal lobe and temporal lobe including hippocampi has been shown, so glutamate and GABA receptors was investigated in these brain regions in present study.

## 2 Material and methods

### 2.1 Animals

Eight-week-old pregnant C57BL/6 mice were purchased from CLEA Japan (Tokyo, Japan). Pregnancy was checked by a vaginal plug. Offspring of pregnant mice given treatment were defined as F1 mice. Male F1 mice were used for the experiment and analysis at 8 weeks of age. Eight-week-old C57BL/6 male mice, purchased from CLEA Japan, were used as acute mice. All animals were housed at 22 ± 2 °C with free access to water and food. All experiments were conducted according to the Guidelines for Animal Experimentation of Ehime University Graduate School of Medicine (Ehime, Japan) and the ethics committee of Ehime University Graduate School of Medicine (No. 28–34).

### 2.2 Administration of drugs and experimental procedures

HAL was purchased from Tokyo Chemical Industry (Tokyo, Japan) and dissolved in normal saline. Offspring of mice that were intraperitoneally injected with HAL during pregnancy at 1 mg/kg/day were the HAL group, and offspring of mice that were intraperitoneally injected with the same volume of normal saline during pregnancy were the control group. HAL and normal saline were administered twice a day (at 07:00 and 18:00 h) from pregnancy day 1 to birth. Offspring were weaned at 4 weeks and then housed three per cage. Acute mice were intraperitoneally injected with 1 mg/kg/day HAL or normal saline for 20 days from 8 weeks after birth. F1 mice at 8 weeks of age and acute mice after 20 days of administration were sacrificed by decapitation, and their hippocampi, frontal lobes, and temporal lobes were collected. Brain tissue was immediately stored at − 80 °C until analysis. This procedure was conducted according to the “Guidelines for the Care and Use of Mammals in Neuroscience and Behavioral Research (National Research Council (US) Committee on Guidelines for the Use of Animals in Neuroscience and Behavioral Research 2003)”.

### 2.3 RNA isolation, synthesis of complementary DNA (cDNA), and reverse transcription-quantitative PCR (RT-qPCR)

RNA was isolated according to the previously reported protocol (Roy et al., 2017). TRIzol® (Thermo Fisher Scientific, Carlsbad, CA, USA) was used for RNA isolation. RNA concentration and quality were calculated using the NanoDrop 1000 system (Thermo Fisher Scientific, Yokohama, Japan).

Synthesis of cDNA was performed using the High-Capacity cDNA Reverse Transcription Kit (Applied Biosystems, Foster City, CA, USA). RNA (1.0 μg) was used in 40-μl reaction mixtures to synthesize cDNA.

The measurement of mRNA expression levels was conducted by RT-qPCR using the StepOnePlus Real-Time PCR System (Applied Biosystems, Waltham, MA, USA). The Predesigned qPCR Assay used Mm.PT.58.8802331 for *Gria2*, Mm.PT.58.35498238 for *Gria4*, Mm.PT.58.31353856 for *Grik3*, Mm.PT.58.13771721 for *Grin2a*, Hs.PT.58.33686329 for *Grin3a*, Mm.PT.58.32797820 for *Gabra1*, Mm.PT.58.30962343 for *Gabra2*, Mm.PT.58.11312618 for *Gabra3*, and Mm.PT.39a.1 for *Gapdh*. RT-qPCR was conducted using the PrimeTime Gene Expression Master Mix (Integrated DNA Technologies, Inc., Coralville, IA, USA) in a final volume of 10 μl. The mRNA expression levels were measured in duplicate. Relative expression was calculated using the difference between the target gene and *Gapdh*.

### 2.4 Western blotting

Western blotting was performed using the frontal lobes of F1 mice. Brain tissue was homogenized in 500 μl 1xRIPA buffer (FUJIFILM Wako Pure Chemical Corporation, Osaka, Japan) including 50 μl of protease inhibitor (P8340; Sigma-Aldrich Japan, Tokyo, Japan). Homogenates were centrifuged at 12,000 rpm for 20 min at 4 °C, and the supernatant was collected as protein lysate. The protein concentration was measured with a BCA Protein Assay Kit (TaKaRa, Shiga, Japan) using the Flex Station®3 (Molecular Devices Japan, Tokyo, Japan). The protein lysates (15 μg) were separated by 10% SDS-polyacrylamide gels, transferred to membranes using the iBlot Transfer Stacks (Thermo Fisher Scientific, Waltham, MA, USA). After blocking with 5% skim milk (FUJIFILM Wako Pure Chemical Corporation), the membrane was incubated with Anti-Grik3/GluK3 antibody (cat. no. ab183035; Abcam [Cambridge, UK]) diluted 2000 times by TBS-T overnight. The membrane was washed by TBS-T and then treated with goat anti-rabbit (H+J) (Jackson Immunoresearch, West Grove, PA, USA.) diluted 2000 times by TBS-T at room temperature for 1 h. After stripping the antibody with stripping buffer composed of 0.2 M glycine, 1 % Tween20, 0.1 % SDS, and Milli-Q water and blocking, the membrane was incubated with Anti-Gapdh antibody (cat. no. ab22555; Abcam) diluted 2000 times by TBS-T overnight. The membrane was washed by TBS-T and then treated with goat anti-rabbit (H+J) diluted 2000 times by TBS-T. Visualization using ChemiDoc MP (Bio Rad) was used for detection. Image J (http://rsb.info.nih.gov/ij/index.html) was used for digitization and statistical analysis.

### 2.5 Statistical analysis

The statistical analysis was performed with SPSS 22.0 software (IBM Japan, Tokyo, Japan). Analysis of the normality of distributions was performed using the Shapiro-Wilk test, and average differences in mRNA expression levels and Western blots between two groups were assessed using Student’s *t*-test or the Mann–Whitney U test. A p value < 0.05 was regarded as significant.

## 3 Results

### 3.1 mRNA expression levels of F1 mice

**Fig. 1:**
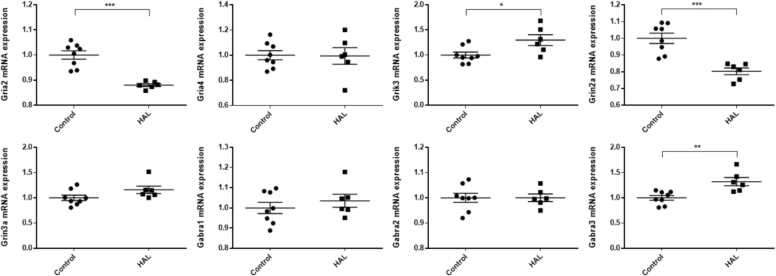
The mRNA expression levels in the hippocampi of F1 mice of the haloperidol and control groups. HAL: Offspring of mice intraperitoneally injected with HAL during pregnancy at 1 mg/kg/day from pregnancy day 1 to birth. Control: Offspring of mice that were intraperitoneally injected with normal saline during pregnancy at 1 mg/kg/day from pregnancy day 1 to birth. Average differences in mRNA expression levels between the two groups were assessed by Student’s *t*-test or the Mann–Whitney U test. The data are shown as *p < 0.05, * *p < 0.01, * **p < 0.001. n = 6 for the HAL group and n = 8 for the control group.

**Fig. 2:**
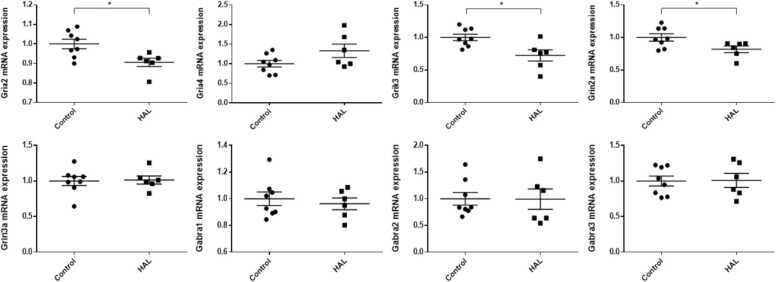
The mRNA expression levels in the frontal lobes in the haloperidol and control groups of F1 mice. HAL: Offspring of mice that were intraperitoneally injected with HAL during pregnancy at 1 mg/kg/day from pregnancy day 1 to birth. Control: Offspring of mice that were intraperitoneally injected with normal saline during pregnancy at 1 mg/kg/day from pregnancy day 1 to birth. Average differences in mRNA expression levels between the two groups were assessed by Student’s *t-*test or the Mann–Whitney U test. The data are shown as ∗p < 0.05. n = 6 for the HAL group and n = 8 for the control group.

**Fig. 3:**
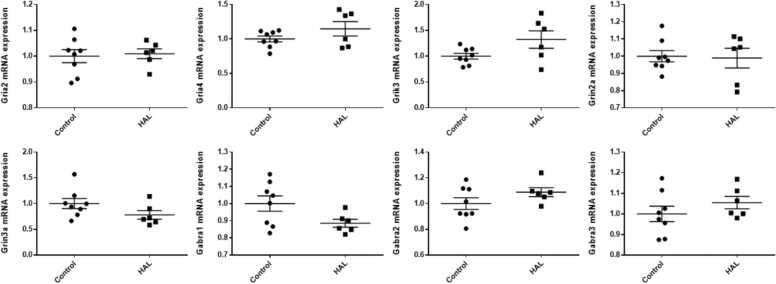
The mRNA expression levels in the temporal lobes of the haloperidol and control groups of F1 mice. HAL: Offspring of mice that were intraperitoneally injected with HAL during pregnancy at 1 mg/kg/day from pregnancy day 1 to birth. Control: Offspring of mice that were intraperitoneally injected with normal saline during pregnancy at 1 mg/kg/day from pregnancy day 1 to birth. Average differences in mRNA expression levels between the two groups were assessed by Student’s *t-*test or the Mann–Whitney U test. n = 6 for the HAL group and n = 8 for the control group.

In the hippocampi, *Grik3* and *Gabra3* mRNA expression levels were significantly higher in the HAL group than in the control group (*Grik3:* p = 0.023, *Gabra3*: p = 0.004), whereas *Gria2* and *Grin2a* mRNA expression levels were significantly lower (*Gria2*: p < 0.001, *Grin2a*: p < 0.001) in the HAL group than in the control group (Fig. 1). There were no significant differences in *Gria4*, *Grin3a*, *Gabra1*, and *Gabra2* mRNA expression levels in the hippocampi and in *Gria4*, *Grin3a*, *Gabra1*, *Gabra2*, and *Gabra3* mRNA expression levels in the frontal lobes. *Gria2*, *Grik3*, and *Grin2a* mRNA expression levels were significantly lower in the HAL group than in the control group in the frontal lobes (*Gria2*; p = 0.015, *Grin2a*; p = 0.037, *Grik3*; p = 0.012) (Fig. 2). For all of the genes examined in the present study, there were no significant differences in mRNA expression levels in the temporal lobes (Fig. 3).

### 3.2 mRNA expression levels of acute mice

**Fig. 4:**
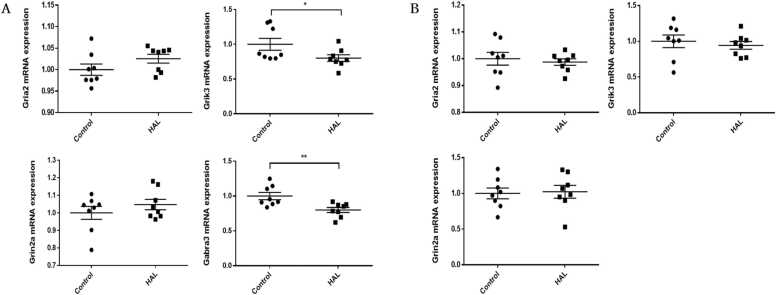
The mRNA expression levels in (A) the hippocampi and (B) frontal lobes of the haloperidol and control groups of acute mice. HAL: Male mice that were intraperitoneally injected with 1 mg/kg/day HAL for 20 days from 8 weeks after birth. Control: Male mice that were intraperitoneally injected with 1 mg/kg/day normal saline for 20 days from 8 weeks after birth. Average differences in mRNA expression levels between the two groups were assessed by Student’s *t*-test or the Mann–Whitney U test. The data are shown as ∗p < 0.05, ∗∗p < 0.01. n = 8 for each group.

To confirm whether the changes of mRNA expression levels in F1 mice were related to HAL exposure during pregnancy, the expression levels of significantly changed genes in F1 mice were measured in the hippocampi and frontal lobes of acute mice. In the hippocampi, *Grik3* (p = 0.049) and *Gabra3* (p = 0.007) mRNA expression levels were significantly decreased in the HAL group, whereas *Gria2* and *Grin2a* mRNA expression levels were not significantly different (Fig. 4A). In the frontal lobes of acute mice, there were no differences in *Gria2*, *Grin2a*, and *Grik3* mRNA expressions levels (Fig. 4B).

### 3.3 Western blot of GRIK3

**Fig. 5:**
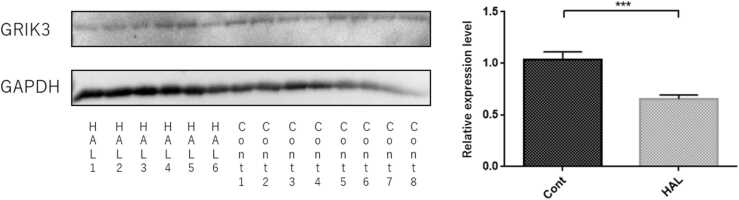
The protein expression levels of GRIK3 in the frontal lobes of F1 mice measured by Western blotting. HAL 1–6: Offspring of mice that were intraperitoneally injected with HAL during pregnancy at 1 mg/kg/day from pregnancy day 1 to birth. Control 1–8: Offspring of mice that were intraperitoneally injected with normal saline during pregnancy at 1 mg/kg/day from pregnancy day 1 to birth. The protein expression levels of glutamate receptor, ionotropic, kainate 3 (GRIK3) (104 kDa), and glyceraldehyde 3-phosphate dehydrogenase (GAPDH) (36 kDa) were evaluated by Western blotting. Average differences of GRIK3 and GAPDH between the two groups were assessed by the Mann–Whitney U test. The data are shown as means ± SEM. ∗∗∗p < 0.001.

Since changes in Grik3 mRNA expression levels were seen in the hippocampi and frontal lobes of F1 mice and the hippocampi of acute mice, GRIK3 protein expression was evaluated, and it was significantly lower in the frontal lobes in the HAL group than in the control group (p < 0.001) (Fig. 5).

## 4 Discussion

To the best of our knowledge, this is the first study to investigate the effects of intraperitoneal HAL injection to pregnant mice on the mRNA and protein levels related to glutamate and GABA receptors in the brains of their offspring. There were two major findings.

First, intraperitoneal HAL injection during pregnancy affected mRNA expression related to glutamate receptors in the brain of offspring. *GRIA2* is a gene that encodes one of the subunits constituting the α-amino-3-hydroxy-5-methyl-4-isoxazole propionic acid receptor. The T/T genotype of *GRIA2* was reported as a low-risk genetic marker of paranoid schizophrenia (Gareeva and Khusnutdinova, 2014). In rhesus macaques, six months of HAL administration increased *GRIA2* mRNA expression levels in the dorsolateral prefrontal cortex (O'Connor et al., 2007). Regarding the present result, *Gria2* mRNA expression was decreased in both the hippocampi and frontal lobes of F1 mice, whereas there was no change in acute mice. This result may suggest that decreased *Gria2* mRNA expression was a result of negative feedback via temporary stimulation by HAL during pregnancy. *Grin2a*, which encodes one of the glutamate NMDA receptor subunits, the epsilon-1 subunit of the NMDA receptor, is known as the epilepsy factor (Li et al., 2020, Yang et al., 2018). The reduction of *Grin2a* mRNA expression in the hippocampi and frontal lobes of F1 mice, but not in acute mice, was confirmed. GluN2A, encoded by *GRIN2A*, is reported to be expressed at a low level in the prenatal stage, and it increases at birth (Myers et al., 2019). The difference in *Grin2a* mRNA expression levels between F1 and acute mice may be due to the timing of HAL exposure (F1 mice: from pregnancy day 1 to birth vs. acute mice: 20 days from 8 weeks after birth). The mRNA expression levels of *Grin1* and *Grin2a* were increased, whereas the mRNA expression level of *Grin2b* was decreased with the advance of fetal age, particularly during the second trimester of gestation in the human fetal cerebral cortex (Bagasrawala et al., 2017, Rodenas-Ruano et al., 2012, Sanz-Clemente et al., 2013). Although the mechanism of the varying mRNA expressions of fetal mice and the site specificity in the present results were unclear, the neurotoxicity of HAL during pregnancy might be one factor responsible for these results (Zhuravliova et al., 2007). The mRNA expression of *Grik3*, which encodes the principal subunit of the kainate-type ionotropic glutamate receptor, was also measured in the present study. In the HAL group, the *Grik3* mRNA expression level was significantly increased in the hippocampi of F1 mice and decreased in the frontal lobes of F1 mice and in the hippocampi of acute mice compared with the control group. In addition to the significant change of *Grik3* mRNA expression in the present study, a relationship between *GRIK3* polymorphisms and increased schizophrenia risk was reported in a previous study (Dai et al., 2014). Therefore, GRIK3 protein expression was also evaluated by Western blot in the present study. The significant decrease of the GRIK3 protein level in the frontal lobes of F1 mice in the HAL group might suggest that the reduction of the protein level was caused by decreased *Grik3* mRNA expression. An association between infants born very preterm with an atypical neonatal neurobehavioral profile and differential methylation at the CpG site in GRIK3 in humans has been reported (Everson et al., 2019). Thus, the effect of antipsychotics on Grik3 mRNA and protein expressions may affect the neurobehavioral profile of offspring.

Second, intraperitoneal HAL injection during pregnancy also affected mRNA expression related to GABA receptors in the brains of offspring. The mRNA expression of *Gabra3*, encoding the alpha3 subunit of the GABA(A) receptor, was significantly increased in the hippocampi of F1 mice by intraperitoneal HAL injection during pregnancy. On the other hand, this mRNA expression in the hippocampi of acute mice was significantly decreased in the HAL group compared with the control group. It has been reported that the regulation of *Gabra3* mRNA expression in the hippocampus is related to an antianxiety effect (Sharma et al., 2021). HAL could have an effect on psychosis by regulating *Gabra3* mRNA expression in the hippocampus.

The use of antipsychotics including HAL during pregnancy has been shown to induce teratogenic or embryonal fetotoxic effects (Iqbal et al., 2005). A previous study suggested that administration of HAL to pregnant rats decreased the low-affinity nerve growth factor binding sites and the mRNA expression of nerve growth factor (Alberch et al., 1991). In humans, the amount of placental passage of HAL is about 65 % (Newport et al., 2007). Although the amount of placental passage of HAL in mice is unclear, transplacental exposure should be considered one of the major mechanisms for the effects of intraperitoneally injected HAL on the fetus in the present study. Johnson et al. found that exposure to antipsychotics during pregnancy induced delayed neurodevelopment in humans (Johnson et al., 2012). Since glutamate and GABA receptors mediate important synaptic transmission in the central nervous system, the present results may explain a mechanism of neurodevelopmental modification. As a result of modification by antipsychotics, the effects on cognitive function, perception, and behavior may have appeared through an imbalance of excitation and inhibition in the brain.

This study had several limitations. First, the effects on neurodevelopment and/or behavioral consequences were not examined. In a future study, behavioral tests such as the elevated plus maze test or the open-field test should be conducted in this model. Moreover, the measurement of mRNA in F1 mice was performed only at 8 weeks. To investigate the sequential changes of mRNA expression, mRNA expression changes should be measured at several time points.

## 5 Conclusions

The administration of HAL during pregnancy caused changes in the mRNA expression levels of genes related to glutamate and GABA receptors in the hippocampi and frontal lobes of fetuses. *Grik3* and *Gabra3* mRNA expression levels were decreased in hippocampi with 20 days of intraperitoneal HAL injection. Moreover, in the frontal lobes of offspring, GRIK3 protein levels were decreased. The results of the present study may help clarify the molecular biological mechanism involved in fetal exposure to antipsychotics.

## Ethics approval

All experiments were conducted according to the Guidelines for Animal Experimentation of Ehime University Graduate School of Medicine (Ehime, Japan) and the ethics committee of Ehime University Graduate School of Medicine (No. 28–34).

## Funding

This work was partially supported by a Health and Labour Science Research Grant from the Japanese Ministry of Health, Labour and Welfare and a Grant-in-Aid for Scientific Research from the Japanese Ministry of Education, Culture, Sports, Science and Technology, JSPS KAKENHI Grant Number 20K16628.

## CRediT authorship contribution statement

Hiroshi Kumon and Yuta Yoshino: Conceptualization, Methodology, Software. Hiroshi Kumon and Yuta Yoshino: Data curation, Writing- Original draft preparation. **Yuta Yoshino and Yu Funahashi**: Visualization, Investigation. ***Shinichiro Ochi and Jun-ichi Iga**:* Supervision. Hiroshi Kumon and Yu Funahashi: Software, Validation. Jun-ichi Iga and Shu-ichi Ueno: Writing- Reviewing and Editing.

## Declaration of Competing Interest

No potential conflict of interest relevant to this article was reported.
